# Participation in Regular Physical Activity According to the Type of Disability, Sex, Point of Disability Diagnosis, and Ability to Walk Independently in South Korea

**DOI:** 10.3390/healthcare9081079

**Published:** 2021-08-22

**Authors:** Changok Cho, Wonsang Shin, Sunga Kong

**Affiliations:** 1 Planning and Administration Office, Korea Paralympic Committee (KPC), Seoul 05540, Korea; ok@koreanpc.kr (C.C.); mc0881@koreanpc.kr (W.S.); 2 Department of Clinical Research Design and Evaluation, SAIHST, Sungkyunkwan University, Seoul 06355, Korea; 3 Patient-Centered Outcomes Research Institute, Samsung Medical Center, Seoul 06355, Korea

**Keywords:** physical activity, disabled, disability, intellectual disabilities

## Abstract

This study aimed to compare rates of participation in physical activity according to the type of disability, sex, point of disability diagnosis (congenital vs. acquired), and ability to walk independently. The study involved individuals who were registered as disabled based on the 2020 Sports Survey for the Disabled project of the Korea Ministry of Health and Welfare. Participants (mean age: 49.94 ± 12.35 years) included those with physical disabilities (*n* = 889), visual impairments (*n* = 523), hearing/speech impairments (*n* = 412), intellectual disabilities (*n* = 561), and disabilities associated with brain lesions (*n* = 364). Rates of severe (100%) and congenital disability (65.95%) were highest in the intellectual disability group. Acquired disability was most frequent in the physical disability group (94.71%). The highest frequency of independent walking ability was observed in the hearing/speech impairment group (99.27%). The rate of participation in physical activity was significantly higher in the acquired (odds ratio [OR] = 1.46, 95% confidence interval [CI] = 1.12–1.87, *p* = 0.005) and independent walking (OR = 1.43, 95% CI = 1.11–1.84, *p* = 0.005) hearing/speech impairment groups than in the corresponding physical disability groups after adjusting for age, sex, and severity. Our findings highlight the need to promote physical activity for people with physical and intellectual disabilities based on the factors examined in this study.

## 1. Introduction

The proportion of people with disabilities is increasing worldwide [1], with rates more than doubling in South Korea (Korea hereafter) between 2001 and 2016 [2]. The Centers for Disease Control and Prevention (CDC) defines a disability as any condition of the body or mind (impairment) that makes it more difficult for the persons with the condition to do certain activities (activity limitation) and interact with the world around them (participation restrictions) [3]. Disabilities can reduce general social activity [4] and limit the choice of physical activity [5], which can lead to health problems. An analysis of 2017 data from the National Health Information Database in Korea demonstrated a difference of about 30 years in life expectancy when individuals with the most severe grade of disability were compared to those without disabilities [6]. The risk of developing non-communicable diseases (NCD) is also three times higher in people with than in people without disabilities [3]. Managing healthcare for individuals with disabilities can therefore be complex and time-consuming.

Regular physical activity reduces the incidence of NCDs [7] such as cardiovascular disease [8], diabetes [9], and cancer [10], and it is also associated with reduced mortality [11]. Regular physical activity not only reduces the risk of secondary health problems but also positively affects the quality of life [12]. However, according to the Healthy People 2010 report, only 12% of people with disabilities participated in physical activity for more than five days per week, compared to 16% of people without disabilities [13]. Recent initial guidelines of the World Health Organization (WHO) on physical activity and sedentary behavior for people with disabilities reflect the organization’s strong commitment to comprehensive action expressed in the Agenda and the Global Action Plan 2018–2030 [14]. However, given that secondary health problems are likely to occur if regular physical activity is not maintained, it is necessary to secure basic data that can be used to develop policies and measures that promote physical activity among people with disabilities.

Further, the degree of participation in physical activity among people with disabilities is affected by a multifactorial set of barriers and facilitators that are unique to this population. For example, previous studies have explored the barriers and facilitators of physical activity participation for young people with disabilities from the perspective of these young people and their families [15]. It is important to utilize this information to develop intervention strategies with a greater likelihood of success [16].

Managing health for individuals with disabilities requires the consideration of personalized factors such as sex, race, age, health status, and physical fitness. Some or all of these personal factors can affect the level of engagement in health behaviors [17]. Indeed, previous studies have shown that people with disabilities have less access to healthcare and are more likely to participate in certain health risk behaviors, such as obesity and six chronic disease-related behaviors [18]. However, this relationship is difficult to generalize [19]. Until recently, researchers have focused little attention on physical activities related to health and well-being among adults with disabilities. Particularly, large-scale studies are required to obtain data that can aid in the development of physical activity policies for people with disabilities, as are studies that can expand the findings obtained in specific disability groups to other populations. Such studies will ultimately aid in the development of policies and measures that promote participation in physical activities while considering individual characteristics according to the type of disability [20,21]. Therefore, using data from the national 2020 Sports Survey for the Disabled in Korea, the present study aimed to compare rates of participation in physical activity according to sex, point of disability diagnosis (congenital vs. acquired), and ability to walk independently.

## 2. Materials and Methods

### 2.1. Study Participants

In December 2020, we conducted a study involving people who were registered as disabled (total 2,618,918 persons) based on the 2020 Sports Survey for the Disabled project controlled by the Korea Ministry of Health and Welfare, the Korea Ministry of Culture, Sports, and Tourism, and the Korea Paralympic Committee (www.koreanpc.kr or https://kosis.kr/eng/) on 5 March 2021. The proportion of survey participants was 1,598,754 among the 2,618,918 persons with disabilities registered in the survey. A sample size of 10,000 people was extracted based on the type of disability, degree of disability, and proportional distribution by region. Further, 100% of all responses were complete (*n* = 10,000). However, in this study, we collected and included data on only the adult population aged between 20 and 64 years. Therefore, the participants finally included 2749 people aged between 20 and 64 years. In principle, the survey was conducted via visiting interviews, although video interviews (e.g., Zoom) or detention surveys were conducted simultaneously if necessary. Physical disability (*n* = 889), visual impairment (*n* = 523), hearing/speech impairment (*n* = 412), intellectual disability (*n* = 561), and disability associated with brain lesions (*n* = 364) were noted among the participants. The 2020 Sports Survey for the Disabled was conducted with the ethical approval of the Korea Ministry of Culture, Sports, and Tourism and the Korea Paralympic Committee (Institutional Review Board number: 113020), and all participants provided consent for their involvement in the study. The study was conducted per the principles outlined in the Declaration of Helsinki.

### 2.2. Measurements

#### 2.2.1. Physical Activity

The survey examined whether participants had exercised, the number of exercises performed, and the average exercise time during each session over the past year. The intensity of participation in exercise was classified as light, moderate, or vigorous. Regular physical activity was defined as 150 min or more of moderate to vigorous physical activity per week based on CDC guidelines [7].

#### 2.2.2. Other Variables

Other variables assessed included the following. The first is sex: male or female. The second is the severity of disability—severe or mild—classified in accordance with the criteria for determining the degree of disability of the Korea Ministry of Health and Welfare. The third is the point of disability diagnosis: congenital, developmental, or acquired. Congenital disability represents permanent disabilities in pre-pregnancy, mid-natal, or postpartum processes. Developmental disability usually occurs due to brain lesions, polio, etc., with Grades 1 to 3 being classified as severe, and Grades 4 to 6 being considered mild in Korea. Acquired disability represents mid-term disability: permanent disability due to illness, injury, accident, etc., in the course of growth, such as limbless disability and cervical spine or spinal cord disability. Lastly, the fourth variable is whether there exists an ability to walk independently without auxiliary equipment: yes or no.

### 2.3. Statistical Analysis

Data analysis was performed using STATA version 15.0 (STATA Corp., College Station, TX, USA). All variables are presented as means with standard deviations or as proportions (%). We compared participant characteristics according to the type of disability using one-way analysis of variance, and differences in rates between groups were based on chi-square analysis. Logistic regression analysis was conducted to identify the percentage of individuals participating in regular physical activity based on the type of disability, sex, point of disability diagnosis, and ability to walk independently. Adjusted variables included age, sex, and severity. Statistical significance was set at *p* < 0.05.

## 3. Results

The characteristics of the study participants are listed in Table 1. The study included 1850 male individuals with disabilities (mean age: 49.48 ± 12.48 years) and 899 female individuals with disabilities (mean age: 50.83 ± 11.19). The mean age among all 2749 participants was 49.94 ± 12.35 years. The rate of severe disability was highest in the intellectual disability group (100%), which also exhibited the highest frequency of congenital disability (65.95%). Contrastingly, the frequency of acquired disability (94.71%) was highest in the physical disability group. The highest frequency of independent walking ability was observed in the hearing/speech impairment group (99.27%).

**Figure 1:**
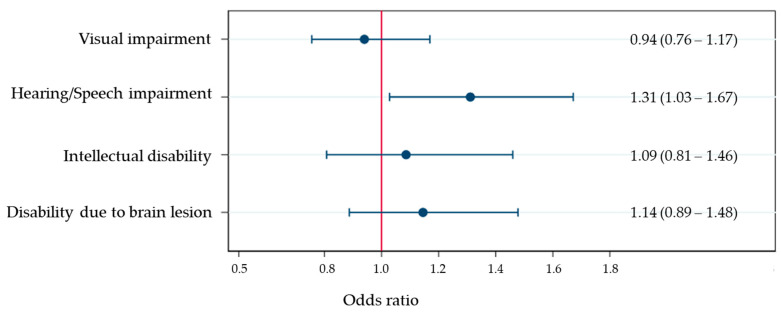
Odds ratios (95% confidence interval) of physical activity according to the type of disability (reference group: physical disability).

When analyses were performed according to the type of disability, the rate of regular participation in physical activity differed significantly between individuals with visual impairments (odds ratio [OR] = 1.31, 95% confidence interval [CI] = 1.03–1.67, *p* < 0.05) compared with individuals with physical disabilities (Figure 1).

When analyses were performed according to sex, separate analyses for male and female indicated that the rate of regular participation in physical activity did not significantly differ between individuals with visual impairments, hearing/speech impairments, intellectual disabilities, or disabilities associated with brain lesions and those with physical disabilities (*p* > 0.05; Table 2).

Analyses based on the point of disability diagnosis revealed that the rate of regular participation in physical activity among the acquired disability group was significantly higher for individuals with hearing/speech impairments (OR = 1.46, 95% CI = 1.12–1.87, *p* = 0.005) than for individuals with physical disabilities. However, rates did not significantly differ between the physical disability group and the visual impairment, intellectual disability, and brain lesion-related disability groups (*p* > 0.05). Furthermore, rates of regular participation in physical activity did not differ between the congenital and acquired disability groups (*p* > 0.05; Table 3).

When analyses were performed based on the ability to walk independently, the rate of participation in regular physical activity was significantly higher in the hearing/speech impairment group (OR = 1.43, 95% CI = 1.11–1.84, *p* = 0.005) than in the physical disability group. However, there were no significant differences between the physical disability group and the visual impairment, intellectual disability, and brain lesion-related disability groups (*p* > 0.05; Table 4).

## 4. Discussion

Using national data for adults with disabilities in Korea, the present study compared rates of participation in physical activity for different types of disabilities based on sex, point of disability diagnosis, and the ability to walk independently. When analyses were performed according to sex, we observed no differences in the rates of physical activity participation. However, participation in physical activity was approximately 45% more likely in individuals with hearing/speech impairments whose conditions were acquired and who were capable of independent walking. This finding highlights the importance of developing measures to promote physical activity based on the type of disability that affects participation in such activity.

In this study, the rate of participation in physical activity among all adults with disabilities was 52.24%. At approximately 60%, this rate was highest among individuals with hearing/speech impairments. However, the intellectual disability group exhibited the lowest participation rate (45%). Generally, people with disabilities are much more inactive than the general population [22,23]. In particular, the 2014 CDC reports indicated that 53.7% of individuals without disabilities engaged in physical activity, but that only around 38.3% of individuals with intellectual disabilities (i.e., cognitive disabilities) did so, consistent with previous findings [24]. Previous studies have shown that both men and women with intellectual disabilities tend to exhibit a sedentary level of physical activity [25], and some prior studies have reported high levels of obesity among trainees attending social education centers [26]. People with intellectual disabilities often experience high levels of nutritional deficiency and dehydration [27], with lower rates of young people participating in sports than the general population [28]. Furthermore, participation in physical activity among those with intellectual disabilities is negatively correlated with age, which may be because older individuals do not generally have the same level of guardian care as younger individuals [29]. While this highlights the important role of the individual’s guardian in promoting physical activity, it also sheds light on the need for programs and policies that focus on physical activity promotion for improving health among those with intellectual disabilities.

In the United States, rates of participation in physical activity (more than 150 min of moderate-intensity aerobic activity per week) for people with disabilities were reported to be 45% for people with hearing impairments, 41% for people with visual impairments, and 21% for people with mobility impairments [24]. In Poland, only 18% of people with mental disabilities and 11% of people with physical disabilities satisfied the WHO recommendations for physical activity [30]. Thus, in some countries, mobility and physical activity are the lowest among individuals with physical disabilities, in contrast to our findings. However, even these areas should focus on the management of physical activity among people with intellectual disabilities, who are known to exhibit lower rates of participation in physical activity than those with hearing/speech impairments and disabilities related to brain lesions.

When participation rates were assessed based on the severity of the disability, a previous study reported that 11.1% and 8.5% of individuals with mild and severe disabilities engaged in the recommended level of activity based on WHO guidelines, respectively [30]. In prior work, Martin Ginis et al. [31] proposed strengthening cooperation among the healthcare, rehabilitation, and community sectors to promote lifelong leisure-time physical activity among individuals with disabilities. We suspect that those with intellectual disabilities exhibited the lowest levels of physical activity in our study because they also had the highest rate of severe disability. Nevertheless, to increase physical activity among the population with disabilities, physical activity approaches should be based on accurate and realistic on-site surveys conducted in appropriately designed facilities, and education regarding physical activity should be provided based on the type of disability.

Low awareness and motivation for exercise and health are the most common reasons why people with disabilities do not lead active lives. In one prior study, many participants cited negative social attitudes toward disabilities as barriers to participation. Participants also noted that some sports and recreational staff and sports leaders lacked experience with people with disabilities [32]. In Poland, 62.5% of people with disabilities participated in sports activities at home and 35.9% outdoors, with only 7.7% of respondents saying they participated in classes within sports clubs or organizations for people with disabilities [30]. This change in the understanding and social awareness of people with disabilities is essential to ensure that people with disabilities have equal access to participation in sports activities. In addition, most rehabilitation centers provide little or no follow-up care to maintain physical activity after the rehabilitation period. Additionally, as reported in a previous study, although the walking speed of athletes with disabilities was similar to that of the general population, it was reported that they had better balance and their fear of falling was slightly higher [33]. Based on these studies, it is possible to promote more effective physical activities if a special-purpose management program alleviating fall-related risk and improving balancing ability, rather than just emphasizing simple physical activity, is provided to people with disabilities.

Therefore, effective strategies to improve and maintain physical functioning and quality of life among individuals with disabilities are required. Promoting the involvement of adults with disabilities in physical activities will require the dedication and cooperation of the public and private organizations responsible for improving each citizen’s health [34].

This study had several limitations. First, it is difficult to interpret our findings as international data because we only examined people with disabilities in Korea. However, since it was an analysis of large-scale survey data conducted in Korea, our findings can be considered representative of the country. Second, since this study was based on surveys, quantitative analysis of physical activity was challenging. Nevertheless, an analysis of survey data from 2749 people is relatively significant. Third, we did not include clinical indicators such as weight, body mass index, general health, health measures, illnesses, or psychosocial factors (the perception, attitudes, expectations, and values regarding engagement in physical activities). Further small-scale cohort studies are required to examine other factors that can influence participation in physical activity among adults with disabilities. Fourth, the 2020 Sports Survey for the Disabled project did not investigate the population of Paralympic athletes but only the population with general disabilities. Fifth, because the 2020 Sports Survey for the Disabled was a cross-sectional retrospective cohort study, cause-and-effect relationships could not be assessed; hence, only the interrelationships between variables were examined. This survey was conducted from November 2020 to January 2021. It was, thus, conducted after the onset of the COVID-19 pandemic, and the results might be different from those we would have obtained before because of the reduced physical activity during the pandemic. Well-designed studies investigating the difference in results before and after the pandemic will be needed in the future.

## 5. Conclusions

Taken together, the results of this study indicate that physical activity is lower among individuals with physical disabilities than among the general population and that those with intellectual disabilities exhibit even lower rates of participation in physical activity than those with physical disabilities. While there were no differences in rates of participation in physical activity according to sex, the rate of participation in physical activity was highest for individuals with hearing/speech impairments whose conditions were acquired and who were capable of independent walking. Ultimately, our findings highlight the need to develop programs and policies that promote physical activity for people with physical and intellectual disabilities based on the type of disability, sex, point of disability diagnosis (congenital vs. acquired), and ability to walk independently.

## Figures and Tables

**Table 1:** Characteristics of study participants.

Group	Total	Physical Disability	Visual Impairment	Hearing/Speech Impairment	Intellectual Disability	Disability Due to Brain Lesion	*p*-Value
Variable	*N* = 2749 (100.00)	*N* = 889 (32.34)	*N* = 523 (19.03)	*N* = 412 (14.99)	*N* = 561 (20.41)	*N* = 364 (13.24)	
Age, year	49.94 ± 12.35	54.33 ± 8.57	51.77 ± 10.36	53.87 ± 9.76	36.99 ± 12.77	52.10 ± 11.33	<0.001 ***
Male (*n* = 1850, 67.30%)	49.48 ± 12.48	53.72 ± 8.71	50.87 ± 10.64	53.68 ± 10.03	35.97 ± 12.70	52.24 ± 11.43	<0.001 ***
Female (*n* = 899, 32.70%)	50.83 ± 11.19	55.88 ± 8.01	53.84 ± 9.42	54.16 ± 9.35	38.86 ± 12.73	51.83 ± 11.19	<0.001 ***
Severity							<0.001 ***
Severe	1119 (40.71)	140 (15.75)	95 (18.16)	154 (37.38)	561 (100)	169 (46.43)	
Mild	1630 (59.29)	749 (84.25)	428 (81.84)	258 (62.62)	0 (0.00)	195 (53.57)	
Meeting physical activity guidelines	1436 (52.24)	474 (53.32)	263 (50.29)	245 (59.47)	253 (45.10)	201 (55.22)	<0.001 ***

Values are represented as the number of participants (%) or as the mean ± standard deviation; *** *p* < 0.001, one-way analysis of variance or chi-square test.

**Table 2:** Odds ratios for physical activity according to the type of disability by sex.

Group Variable	Physical Disability	Visual Impairment	Hearing/Speech Impairment	Intellectual Disability	Disability Due to Brain Lesion
Male (*n*, %)	639 (71.88)	363 (69.41)	248 (60.19)	363 (64.71)	237 (65.11)
Meeting physical activity guidelines (*n*, %)	341 (53.36)	184 (50.69)	144 (58.06)	165 (45.45)	131 (55.27)
Odds ratio (95% CI)	Reference	0.95 (0.73–1.24)	1.21 (0.90–1.63)	1.05 (0.73–1.50)	1.11 (0.82–1.52)
*p*-value		0.718	0.216	0.808	0.503
Female (*n*, %)	250 (28.12)	160 (30.59)	164 (39.81)	198 (35.29)	127 (34.89)
Meet physical activity guidelines (*n*, %)	133 (53.20)	79 (49.38)	101 (61.59)	88 (44.44)	70 (55.12)
Odds ratio (95% CI)	Reference	0.91 (0.61–1.36)	1.50 (0.99–2.27)	1.18 (0.70–1.97)	1.23 (0.78–1.93)
*p*-value		0.648	0.053	0.532	0.368

Values are represented as the number of participants (%). CI, confidence interval. logistic regression analysis adjusted for age and severity.

**Table 3:** Odds ratios for physical activity for each type of disability according to the point of disability diagnosis.

Group Variable	Physical Disability	Visual Impairment	Hearing/Speech Impairment	Intellectual Disability	Disability Due to Brain Lesion
Congenital disability (*n*, %)	47 (5.29)	59 (11.28)	64 (15.53)	370 (65.95)	33 (9.07)
Meeting physical activity guidelines (*n*, %)	25 (53.19)	26 (44.07)	29 (45.31)	164 (44.32)	17 (51.52)
Odds ratio (95% CI)	Reference	0.69 (0.32–1.51)	0.59 (0.26–1.32)	0.69 (0.31–1.52)	1.05 (0.40–2.79)
*p*-value		0.353	0.198	0.360	0.916
Acquired disability (*n*, %)	842 (94.71)	464 (88.72)	348 (84.47)	191 (34.05)	331 (90.93)
Meeting physical activity guidelines (*n*, %)	449 (53.33)	237 (51.08)	216 (62.07)	89 (46.60)	184 (55.59)
Odds ratio (95% CI)	Reference	0.96 (0.76–1.21)	1.46 (1.12–1.87)	1.14 (0.77–1.68)	1.14 (0.87–1.48)
*p*-value		0.739	0.005 **	0.595	0.378

Values are represented as the number of participants (%). CI, confidence interval. ** *p* < 0.01, logistic regression analysis adjusted for age, sex, and severity.

**Table 4:** Odds ratios for physical activity for each type of disability according to the ability to walk independently.

Group Variable	Physical Disability	Visual Impairment	Hearing/Speech Impairment	Intellectual Disability	Disability Due to Brain Lesion
Ability to walk independently (*n*, %)	789 (88.75)	472 (90.25)	409 (99.27)	545 (97.15)	236 (64.84)
Meeting physical activity guidelines (*n*, %)	408 (51.71)	234 (49.58)	245 (59.90)	248 (45.5)	130 (55.08)
Odds ratio (95% CI)	Reference	0.97 (0.77–1.23)	1.43 (1.11–1.84)	1.18 (0.85–1.64)	1.23 (0.91–1.65)
*p*-value		0.829	0.005 **	0.318	0.185

Values are represented as the number of participants (%). CI, confidence interval. ** *p* < 0.01, logistic regression analysis adjusted for age, sex, and severity.

## Data Availability

Data that support the findings of this study are openly available from the Korea Ministry of Health and Welfare, the Korea Ministry of Culture, Sports, and Tourism, and the Korea Paralympic Committee at https://www.koreanpc.kr or https://kosis.kr/eng/ on 5 March 2021.

## References

[1] World Health Organization (2011). World Report on Disability 2011.

[2] Korea Disabled People’s Development Institute (2018). Annual Report on Disability Statistics 2018.

[3] Centers for Disease Control and Prevention Increasing Physical Activity among Adults with Disabilities. Disability and Health 2016. http://www.cdc.gov/ncbddd/disabilityandhealth/pa.html.

[4] Isaksson G., Skär L., Lexell J. (2005). Women’s perception of changes in the social network after a spinal cord injury. Disabil. Rehabil..

[5] Cavill N., Kahlmeier S., Racioppi F. (2006). Physical Activity and Health in Europe: Evidence for Action.

[6] Bahk J., Kang H.-Y., Khang Y.-H. (2019). The life expectancy gap between registered disabled and non-disabled people in Korea from 2004 to 2017. Int. J. Environ. Res. Public Health.

[7] Piercy K.L., Troiano R.P., Ballard R.M., Carlson S.A., Fulton J.E., Galuska D.A., George S.M., Olson R.D. (2018). The physical activity guidelines for Americans. JAMA.

[8] Hegde S.M., Solomon S.D. (2015). Influence of physical activity on hypertension and cardiac structure and function. Curr. Hypertens. Rep..

[9] Bhaskarabhatla K.V., Birrer R. (2005). Physical activity and diabetes mellitus. Compr. Ther..

[10] McTiernan A., Friedenreich C.M., Katzmarzyk P.T., Powell K.E., Macko R., Buchner D., Pescatello L.S., Bloodgood B., Tennant B., Vaux-Bjerke A. (2019). Physical activity in cancer prevention and survival: A systematic review. Med. Sci. Sports Exerc..

[11] Lee I.M., Shiroma E.J., Lobelo F., Puska P., Blair S.N., Katzmarzyk P.T. (2012). Effect of physical inactivity on major non-communicable diseases worldwide: An analysis of burden of disease and life expectancy. Lancet.

[12] Brown D.R., Carroll D.D., Workman L.M., Carlson S.A., Brown D.W. (2014). Physical activity and health-related quality of life: US adults with and without limitations. Qual. Life Res..

[13] Smith B., Kirby N., Skinner B., Wightman L., Lucas R., Foster C. (2019). Infographic. Physical activity for disabled adults. Br. J. Sports Med..

[14] World Health Organization (2020). Guidelines on Physical Activity and Sedentary Behaviour.

[15] Wright A., Roberts R., Bowman G., Crettenden A. (2019). Barriers and facilitators to physical activity participation for children with physical disability: Comparing and contrasting the views of children, young people, and their clinicians. Disabil. Rehabil..

[16] Rimmer J.H., Riley B., Wang E., Rauworth A., Jurkowski J. (2004). Physical activity participation among persons with disabilities: Barriers and facilitators. Am. J. Prev. Med..

[17] World Health Organization (2007). International Classification of Functioning, Disability, and Health: Children & Youth Version: ICF-CY.

[18] McGuire L.C., Strine T.W., Okoro C.A., Ahluwalia I.B., Ford E.S. (2007). Peer reviewed: Healthy lifestyle behaviors among older US adults with and without disabilities, behavioral risk factor surveillance system, 2003. Prev. Chronic Dis..

[19] Pharr J.R., Bungum T. (2012). Health disparities experienced by people with disabilities in the United States: A Behavioral Risk Factor Surveillance System study. Glob. J. Health Sci..

[20] Hoekstra F., Roberts L., van Lindert C., Martin Ginis K.A., van der Woude L.H.V., McColl M.A. (2019). National approaches to promote sports and physical activity in adults with disabilities: Examples from the Netherlands and Canada. Disabil. Rehabil..

[21] Jaarsma E.A., Haslett D., Smith B. (2019). Improving communication of information about physical activity opportunities for people with disabilities. Adapt. Phys. Act. Q..

[22] Steckler A.B., Linnan L., Israel B. (2002). Process Evaluation for Public Health Interventions and Research.

[23] Wierenga D., Engbers L.H., Van Empelen P., Duijts S., Hildebrandt V.H., Van Mechelen W. (2013). What is actually measured in process evaluations for worksite health promotion programs: A systematic review. BMC Public Health.

[24] Carroll D.D., Courtney-Long E.A., Stevens A.C., Sloan M.L., Lullo C., Visser S.N., Fox M.H., Armour B.S., Campbell V.A., Brown D.R. (2014). Vital signs: Disability and physical activity—United States, 2009–2012. Morb. Mortal. Wkly. Rep..

[25] Emerson E. (2005). Underweight, obesity and exercise among adults with intellectual disabilities in supported accommodation in Northern England. J. Intellect. Disabil. Res..

[26] Cole O. (1986). Medical screening of adults at social education centres: Whose responsibility. Mental Handicap.

[27] Macdonald N., McConnell K., Stephen M., Dunnigan M. (1989). Hypernatraemic dehydration in patients in a large hospital for the mentally handicapped. Br. Med. J..

[28] Flynn M., Hirst M. (1992). This Year, Next Year, Sometime...?: Learning Disability and Adulthood.

[29] Robertson J., Emerson E., Gregory N., Hatton C., Turner S., Kessissoglou S., Hallam A. (2000). Lifestyle related risk factors for poor health in residential settings for people with intellectual disabilities. Res. Dev. Disabil..

[30] Biernat E., Piatkowska M. (2017). Physical activity of disabled individuals in the context of meeting WHO recommendations and support of local authorities. Turk. J. Phys. Med. Rehabil..

[31] Martin Ginis K.A., Ma J.K., Latimer-Cheung A.E., Rimmer J.H. (2016). A systematic review of review articles addressing factors related to physical activity participation among children and adults with physical disabilities. Health Psychol. Rev..

[32] Shields N., Synnot A. (2016). Perceived barriers and facilitators to participation in physical activity for children with disability: A qualitative study. BMC Pediatrics.

[33] da Silva E.S., Fischer G., da Rosa R.G., Schons P., Teixeira L.B.T., Hoogkamer W., Peyré-Tartaruga L.A. (2018). Gait and functionality of individuals with visual impairment who participate in sports. Gait Posture.

[34] Centers for Disease Control and Prevention (2007). Physical activity among adults with a disability—United States, 2005. Morb. Mortal. Wkly. Rep..

